# Spatially-explicit models of global tree density

**DOI:** 10.1038/sdata.2016.69

**Published:** 2016-08-16

**Authors:** Henry B. Glick, Charlie Bettigole, Daniel S. Maynard, Kristofer R. Covey, Jeffrey R. Smith, Thomas W. Crowther

**Affiliations:** 1 Yale School of Forestry and Environmental Studies, Yale University, New Haven, CT 06511, USA; 2 Center for Conservation Biology, Department of Biology, Stanford University, Stanford, CA 94305, USA; 3 Netherlands Institute of Ecology, Wageningen 6700 AB, Netherlands

**Keywords:** Ecological modelling, Forest ecology, Forestry, Macroecology

## Abstract

Remote sensing and geographic analysis of woody vegetation provide means of evaluating the distribution of natural resources, patterns of biodiversity and ecosystem structure, and socio-economic drivers of resource utilization. While these methods bring geographic datasets with global coverage into our day-to-day analytic spheres, many of the studies that rely on these strategies do not capitalize on the extensive collection of existing field data. We present the methods and maps associated with the first spatially-explicit models of global tree density, which relied on over 420,000 forest inventory field plots from around the world. This research is the result of a collaborative effort engaging over 20 scientists and institutions, and capitalizes on an array of analytical strategies. Our spatial data products offer precise estimates of the number of trees at global and biome scales, but should not be used for local-level estimation. At larger scales, these datasets can contribute valuable insight into resource management, ecological modelling efforts, and the quantification of ecosystem services.

## Background & Summary

In this paper we detail the background, methods, and data associated with the first spatially-continuous model of global tree density^1^. This research was motivated by (i) a gap in the publically available forest-based geospatial data products, (ii) a specific request from Plant for the Planet Foundation, and (iii) recently published estimates of tree density in the Amazon basin^2^ implying that the previous estimate of the number of trees globally^3^ was potentially an order of magnitude too low.

Forests cover approximately one-third of the world’s terrestrial land surface^4^. They are fundamental in dictating ecosystem structure^5,6^, biogeochemical processes^7,8^, animal habitat^9^, biomass and carbon sequestration^10,11^, and anthropogenic demand for building materials, pulp products, and fuelwood^12^. An understanding of the extent of forest resources plays a critical role in sustainable forest management^4^, helping to guide policy and to provide key targets for initiatives like the Convention on Biological Diversity’s Strategic Plan for Biodiversity 2011–2020 (ref. 13), the United Nations Collaborative Programme on Reducing Emissions from Deforestation and Forest Degradation in Developing Countries (REDD)^14^, and the landmark 2015 United Nations Conference of Parties Agreement^15–17^.

A number of recent studies inform our understanding of the distribution and extent of forest resources^2,18–21^. However, until recently, global scale models have not focused on estimating forest population parameters such as total tree numbers or tree density^1^. These variables complement existing data (e.g., refs 19,22–24) and lend themselves to modelling biogeochemical processes^8,25^, nutrient cycling^26^, habitat suitability^9^, forest biodiversity^27^, and drivers of forest structure and heterogeneity^28,29^. Furthermore, the number and density of trees are intuitive metrics of interest to public and non-governmental organizations^30,31^, particularly those focused on tree planting, such as New York City’s MillionTreesNYC^32^ and the United Nations Environment Programme’s (UNEP) ‘Billion Tree Campaign’^33^.

To quantify the proportional impact of these reforestation campaigns and to establish meaningful reforestation targets, a baseline understanding of current tree numbers was essential. We initially developed the global tree density analysis to address this uncertainty. Further, we hypothesized that available data would demonstrate the extent to which biophysical and social variables interact to regulate global patterns in tree abundance. The previous estimate for the global number of trees was approximately 400.25 billion^3^—a mere 10 billion more than has been estimated for the Amazon basin during a recent broad-scale inventory^2^—highlighting a critical gap in our understanding of global tree densities.

We emphasize that the modelling approach described herein provides precise estimates of total number of trees and mean tree density at global and biome scales. However, this precision does not necessarily apply to smaller scales and our data products should not be used for local- or regional-scale analysis without further assessment (see Usage Notes).

## Methods

### Overview

**Figure 1:**
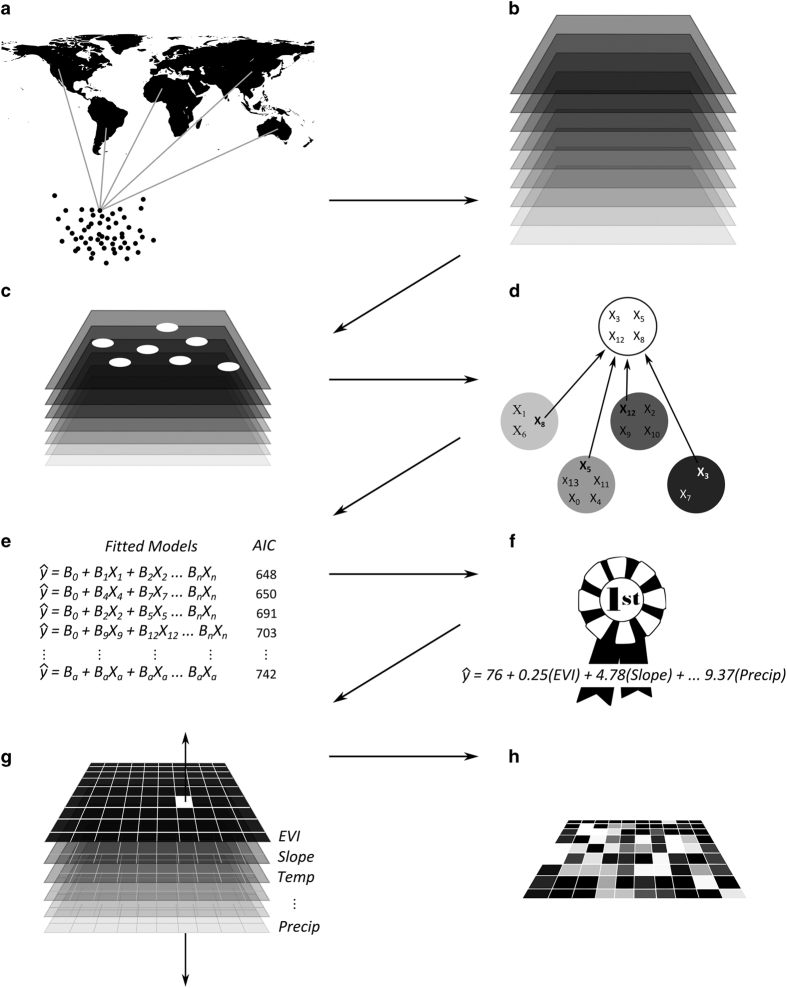
A conceptual model of our analytical process. (**a**) We amassed over 420,000 forest inventory plot records from every continent except Antarctica. (**b**) We acquired and unified an initial pool of four-dozen spatial covariates to use in model development. (**c**) We selected a subset of spatial covariates, extracted their values at field plot locations, and bound these values to the plot records. (**d**) For each of 14 biomes we subjected the enhanced plot records to hierarchical (agglomerative) clustering to identify the least collinear collection of covariates. (**e**) Generalized linear models were fit to every possible combination of clustered covariates. (**f**) A top ranking predictive model was selected or created through model averaging. (**g**) Each biome’s top ranking model was applied in a pixel-level map algebraic framework. (**h**) We scaled a penultimate spatial model of tree density using land cover data to arrive at our final predictions.

To model global tree density we employed a spatially-explicit approach in which (i) field measurements were first linked to a suite of remote sensing and GIS covariates; (ii) predictive regression models were then developed using model selection criteria; and (iii) these models were then applied in a pixel-level map algebraic framework to develop spatially-explicit predictions of global tree density (Fig. 1).

### Data collection and standardization

To model tree density across large geographic extents we collected field-based forest inventory plot records from around the globe. Plot-level data was obtained through three channels: (i) major forestry databases, (ii) peer-reviewed studies, and (iii) correspondence with individual scientists. We used both national and international forestry databases, including National Forest Inventory (NFI) analyses from 21 countries, the Global Index of Vegetation-Plot Database (GIVD http://www.givd.info), the Smithsonian Tropical Research Institute’s in-house database (http://www.stri.si.edu), and ICP-Level-I plot data for most of Europe (http://www.icp-forests.org). These sources provided the vast majority of our data, but were supplemented with inventory data reported through peer-reviewed publications during the last 10 years^2,34,35^. Small, unpublished collections of field data were obtained through several contacts where we lacked broad-scale inventory data (P. Umunay, DRC; R. Tavani, European NFI).

Although we defined trees as those larger than 10 cm diameter at breast height (DBH; i.e., to separate established trees from seedlings and saplings), the minimum-diameter thresholds for what constitutes a tree vary by country and inventory purpose. In the U.S. NFI—the Forest Inventory and Analysis National Program (FIA)—a tree is defined as a plant with a woody stem and DBH equal to or greater than 12.7 cm (i.e., 5 in). However, the 10 cm DBH threshold is used across most international forest inventory analyses, and the U.S. FIA was easily adapted to this level. After threshold identification, plot data was cleaned and collated using R (v. 3.1.x, Core R Development Team 2015), providing a total of 429,775 independent records for which we had, at a minimum: (i) latitude, (ii) longitude, and (iii) tree density (trees per hectare). Density measurements were derived through a number of proven field methods, including both fixed and variable radius plot sampling.

In the U.S., FIA data had been subjected to ‘jiggering’ or ‘fuzzing’, and ‘swapping’, in which plot locations had been spatially relocated by the US Forest Service to prohibit direct knowledge of plot locations^36,37^. A jiggered plot is generally less than 0.5 mi, but up to 1 mi, from the original plot location, and is to be placed in a stand that shares comparable structural attributes (i.e., the density of a jiggered plot must match the density of its true plot). McRoberts *et al.*^36^ suggest that plot jiggering should not introduce extensive bias in regression modelling when working with large pixel sizes (here ~1 km^2^), large sample sizes, and across broad spatial extents (see *Acquisition and pre-processing of spatial data*). Swapping describes the swapping of up to 20% of private plot coordinates between comparable plots within the same county, making local-level estimate impossible. Neither jiggering nor swapping should have affected our results given the spatial extents of our models.

### Acquisition and pre-processing of spatial data

To generate spatially-explicit estimates of global tree density, we first acquired or developed 48 map-based covariates to consider during model development (Table 1 (available online only)). These datasets were pre-processed using R’s ‘raster’ package^38^, ArcMap 10.1 (ESRI, Redlands, CA), and conventional spatial data management strategies, including, as necessary: mosaicking raster tiles and unifying projections, ensuring precise pixel-level spatial coincidence using environmental processing controls and nearest neighbor resampling, using map algebraic operators and ArcGIS geoprocessing tools to create derivatives, performing spatial extractions (masking) to ensure a common spatial extent across datasets, and performing spatial joins to extract covariate values (Table 2). Each of the 48 datasets was obtained and initially managed using the World Geodetic System 1984 (WGS84) at a spatial resolution of 30-arc seconds (0.008333 degrees). Covariate pixel values were bound to spatially coincident field plot locations stored in a vector point file, ultimately producing a single tabular dataset from which to generate statistical models. During preliminary data exploration we discarded 28 less useful covariates based on (i) multicollinearity and (ii) mismatches between spatial resolution and scale. The remaining 20 covariates captured a range of topographic, climatic, vegetative, and anthropogenic factors (Table 1 (available online only)). We present the full complement of covariates and their primary sources, including those omitted in final analyses, to provide a clear sense of the data considered throughout model development.

Given the inherent variability of plot-based tree density estimates, we developed spatial models for large geographic extents ensuring a high degree of confidence in mean tree density estimates. We relied on two maps of ecologically unique regions delineated by The Nature Conservancy (TNC): Biomes and Ecoregions (Terrestrial Ecoregions map - http://maps.tnc.org/gis_data.html). Biomes are large geographic areas (i.e., continental scale) linked through similarities in biodiversity and associated drivers, originally developed by the World Wildlife Fund (WWF; www.worldwildlife.org/biomes) as the Terrestrial Ecoregions component of their Global Ecoregions dataset. TNC’s Terrestrial Ecoregions are smaller, more localized regions that share a similar habitat type. Individual predictive models were developed for each of the 14 unique terrestrial biomes and 806 unique ecoregions that possibly contained forested land. These two models—hereafter biome-level and ecoregion-level—were used to create two estimates of global forest density corresponding to two different spatial scales of inquiry.

### Statistical modelling

Using the above-mentioned tabular data, we produced statistical models through a multi-step process: (i) hierarchical clustering; (ii) model selection; (iii) model pairing. Given the interactive nature of many biophysical factors, we suspected strong interactions and/or multicollinearity among the selected set of 20 variables (Table 1 (available online only)). To account for this we used ascendant (agglomerative) hierarchical clustering for each biome-level model. Hierarchical clustering is an unsupervised learning algorithm in which groups of values are iteratively split and merged, ultimately dividing them into clusters whose inter-group distance or within-group homogeneity is maximized^39^. In this way, the strategy is similar to other iterative clustering algorithms such as ISODATA or k-means^40^. The product of clustering is a series of discrete groups (clusters) that contain values from one or more covariates that are better correlated with one another than with covariates in another cluster.

We employed ascendant hierarchical clustering using the *hclustvar* function in the *ClustOfVar* R package^41^. Homogeneity of clusters was defined as the sum of the squared correlation between the variables in a cluster and their respective cluster center (here a synthetic quantitative variable equivalent to the first principal component of a PCA mix analysis). To maximize predictive model strength and reduce collinearity we selected a single best ‘indicator’ variable from each cluster based on the squared loading values. This process produced, for each set of field plot records associated with a given biome or ecoregion, a single set of top-ranking covariates to consider during model development.

To estimate tree density in each biome we used the reduced set of covariates to construct generalized linear regression models^42^ with a negative binomial error structure (to accommodate count data that cannot extend below zero). To optimize model strength we used *dredge*, a multi-model dredging function in R’s *MuMln* package^43^. This function evaluates and ranks all possible candidate models from a set of predictors in a global model according to Akaike Information Criterion (AICc) and AIC likelihood weights (AICw). Where no single covariate was overwhelmingly influential, there were, in most cases, a number of candidate models nested within the global model that performed comparably well. We therefore employed weighted model averaging of the dredged models with cumulative AIC weights≥0.95 (ref. 44).

We constructed a unique regression model for each biome or ecoregion that contained at least 50 tree density measurements (for rationale see Model validation and testing). We lacked sufficient plot data for two of the forested biomes: ‘Mangroves’ and ‘Tropical and subtropical coniferous forests’, primarily due to the relative rarity of these biomes worldwide (representing 0.23 and 0.48% of the global land surface, respectively). In both cases we used models from the most analogous biomes for which we had sufficient data, relying on similarity in geography and general ecological conditions (e.g., moist environment, broadleaf species). The ‘Tropical and subtropical moist broadleaf’ biome was substituted for the ‘Mangroves biome’, and ‘Temperate coniferous biome’ for the ‘Tropical and subtropical coniferous’ biome. Because we used ecological analogy, the biome-level estimates for these areas should be considered less reliable than those of other biomes. At the ecoregion level, the distribution of plot-level data prevented us from modelling a large number of global ecoregions. For each of the missing ecoregion models we used the spatially coincident biome-level model in its place, such that the final global ecoregion model of tree density is largely driven by biome-level regression models.

### Spatial modelling

Our final biome- and ecoregion-level negative binomial regression models were applied in a map algebraic framework^45^ using an iterative looping structure in R. We relied on the *doSnow* and *foreach* packages^46,47^ to perform computations in an embarrassingly parallel manner, such that each computational task bore no dependency on any other computational task. For both models, random access memory (RAM) limitations were bypassed by individually processing more than 10,000 geographically distinct regions and mosaicking the results to create a final map of predicted global tree density.

**Figure 2:**
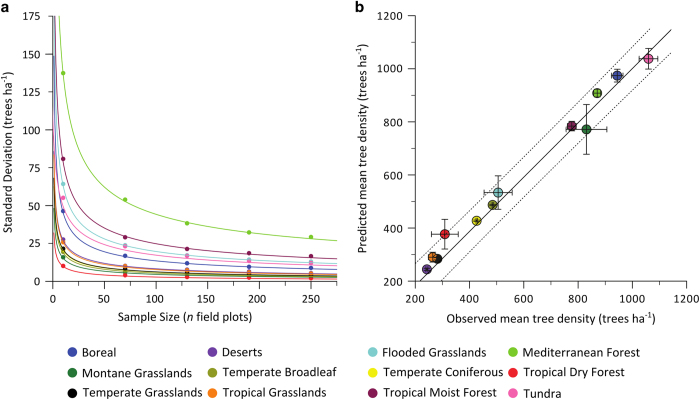
Statistical and spatial model validation. (**a**) The standard deviation of the predicted mean number of trees per biome as a function of sample size. As sample size increases, the variability of the predicted mean tree density reaches a threshold, beyond which an increase in sample size results in a minimal increase in precision. Standard deviations were calculated using a bootstrapping approach (see *Statistical model validation*), and smooth curves were modeled using standard linear regression with a log–log transformation. After Crowther *et al* (2015) Fig. 3b. (**b**) Biome-level regression models predict the mean values of the omitted validation plot measurements in 12 biomes. Overall, the models underestimated mean tree density by ~3% (slope=0.97) but this difference was not statistically significant (*P*=0.51). Bars show±one s.d. for the predicted mean and the dotted boundaries represent the 95% confidence interval for the mean. The values plotted here represent mean densities for the plot measurements (that is, for forested ecosystems), rather than those predicted for each entire biome. Figure is modified from Crowther *et al* (2015) Fig. 3a.

Prior to making area-dependent calculations, mosaicked datasets were reprojected to the Interrupted Goode Homolosine projected coordinate system^48^ and outlying predictions were truncated to 10,000 trees · ha^−1^ based on biome-level variability and expert knowledge of forest structure. Density estimates were then scaled from per-hectare units to per-pixel units where each pixel was nominally 1 km^2^ (897.27 m×897.27 m, or 0.805 km^2^ under Goode Homolosine projection). Since forest reference plots were predominantly located in moderately forested areas (63% +/− 35% [1 s.d.] forested, on average), the original model had minimal predictive power in regions with markedly different land cover types than the reference plots (i.e., in grasslands, deserts, or densely forested areas). To improve the spatial mapping, we therefore used a basic ratio estimation approach^49^ to scale the raw model means by an auxiliary independent data set—the global 1-km consensus land cover data set of 2014 (ref. 21)—which provides an estimate of the percent forested area for each pixel globally. First, we scaled the raw model means by the average percent forest area in the reference plots, on a biome-by-biome basis. We then multiplied this ratio by the percent forested area in the non-reference pixels. This estimation improved the final map characteristics by ensuring that pixels with 0% forests were assigned a value of zero trees, and that the difference in tree totals between two pixels with identical covariate values was directly proportional to their relative difference in percent forest cover. More importantly, this approach ensured that the global and biome-level marginal tree totals were approximately unbiased^49^ (see Fig. 2, Table 3).

### Code availability

We used custom scripting to automate a variety of tasks in the production of our global models of tree density. Covariate pre-processing was partially automated using R (v. 3.1.x, R Core Development Team 2015) and the *raster*^38^ package. Hierarchical clustering and model selection were fully automated using R’s *ClustOfVar*^41^ and *MuMln*^43^ packages, as was the spatial application of statistical models in parallel using the *raster*^38^, *doSnow*^46^, and *foreach*^47^ packages. See the above portions of Methods for additional details. At the time of publication, we have no plans to distribute the scripts used in our analysis.

## Technical Validation

### Statistical model validation

Using two cross-validation schemes to assess the bias and precision of our tree density estimates at plot locations, we evaluated biome-level regression models prior to applying them in a spatial context. In the first scheme, 20% of plot locations within each biome were withheld at random as an independent testing dataset and regression models were generated (dredged) from the remaining 80% using the hierarchically clustered results noted above. These models were then used to predict tree density at the withheld plot locations and the predicted densities were regressed against the observed densities (see Fig. 2 in (ref. 1)). Table 3 provides a summary of model validation results. The 80% models were constructed independently of the full models (see Statistical Modeling), however the additional 20% of plot locations in the full models only reduced bias in relation to the 80% models.

A second validation scheme evaluated the number of field plots required to maximize the precision of density estimates. Following ref. 50, we used a bootstrapping function to evaluate the incremental decrease in standard deviation of our density estimates as a function of sample size for each biome. From the 20% pool of withheld plots noted above, we used simple random sampling with replacement^51^ to obtain a sample of size *n* (*n*=10, 20, …, 500 plots). We next applied the fitted regression models from the retained 80% of plots to the sample to model density at the *n* omitted plots, from which we computed and stored the standard deviation of estimated densities. To obtain a reliable estimate of the standard deviation of tree densities for each *n,* this process was repeated 10,000 times for each sample size. We then plotted standard deviation as a function of sample size to evaluate the point at which an increase in the number of field plots no longer increased the precision of estimated densities (Fig. 2a). Beyond 50 field plots the inclusion of additional data produced only minor increases in precision. This led us to use 50 field plots as the threshold for whether or not to develop a unique regression model for a given geographic area (see Statistical modelling).

### Spatial model validation

**Figure 3:**
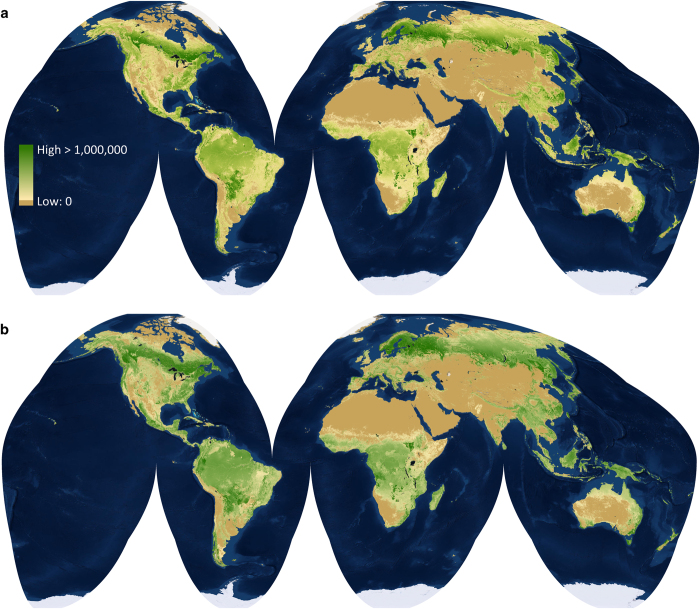
Global models of tree density. Tree density as portrayed through biome- (**a**) and ecoregion-level (**b**) models where values represent number of trees per ~1 km^2^ pixel. Actual pixel size, 897.27 m by 897.27 m in the Goode Homolosine projection. All computations based on areal measurements were made using Goode Homolosine. Maps were produced using ESRI basemap imagery.

**Figure 4:**
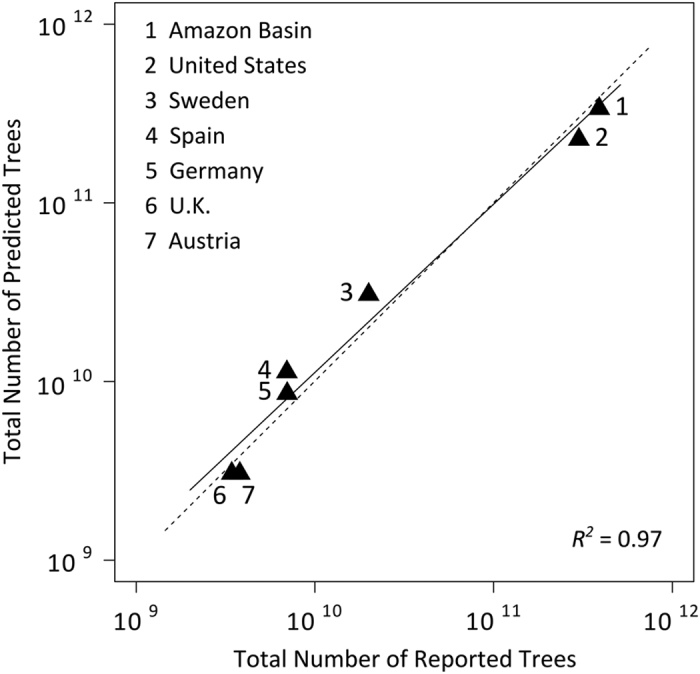
Correlation of predicted and published numbers of trees per country. The dotted line is a 1:1 line, while the solid line is the ordinary least squares line of best fit. Figure is modified from Crowther *et al* (2015) Fig. 4d.

To evaluate the effect of scale on global predictions, spatial models were generated at both the biome and ecoregion levels (Fig. 3). Where our models were sensitive to the proportion of forested land cover within each pixel, we generated independent biome-level models using the consensus land cover dataset^21^ and the map of Global Forest Change 2000–2013 (ref. 19). The former was available at 1 km^2^ spatial resolution while the latter required spatial aggregation from 30 m^2^ to 1 km^2^ to make it compatible with our models. We also compared our predicted tree densities to published country-scale estimates to ensure agreement (Fig. 4).

The close agreement of the biome- and ecoregion-level global models of tree density led us to compute margins of error associated with the biome-level model, which we believed to be more robust given the broader geographic regions over which it was built. We used a Taylor series approximation to estimate the variance in the global and biome-specific totals, accounting for collinearity among predicted values and the log-link negative binomial regression structures (Fig. 2b, Table 4).

## Data Records

Due to data sharing agreements, we are unable to provide direct access to the forest inventory plot data used in model development. However, biome- and ecoregion-level spatial models (maps) of tree density can be downloaded from two locations (see Data Citation). Yale University’s *EliScholar* repository record (Data Citation 1) does not contain a formal DOI at the time of publication and was released with Crowther *et al* (2015) under a CC BY-ND license that prohibits distribution of derivatives made with the data. The *Figshare* repository record (Data Citation 2) offers the same data under a CC-BY license, which permits distribution of derivatives. The authors intend to maintain both repositories in parallel, but refer the reader to the *Figshare* repository as the primary source of new versions of these datasets.

## Usage Notes

To increase the number of field plots within each ecologically-meaningful region, and to account for local-level variability in vegetative structure, we modeled tree density at large spatial scales. Through this approach we were able to obtain high precision in our global estimates of mean tree density and total number of trees. However, it is important to note that precision decreases with a concomitant reduction in spatial extent. Both biome- and ecoregion-level models are less accurate than their global counterparts, country-level estimates are less accurate than their biome- and ecoregion-level counterparts, and pixel-level estimates are less accurate than all other scales of estimation. With this in mind, we are explicit in stating that the spatial models we present here are not intended to provide accurate or precise estimates of tree density at the 1 km^2^ scale, nor even at the scale of small countries.

Spatial models are presented without restriction. See Data collection and standardization for known limitations in field plot information.

## Additional Information

**How to cite this article:** Glick, H. B. *et al.* Spatially-explicit models of global tree density. *Sci. Data* 3:160069 doi: 10.1038/sdata.2016.69 (2016).

## Supplementary Material



## Figures and Tables

**Table 1:** All spatial covariate datasets considered and/or used in analysis, as well as their sources and processing notes

**Layer**	**Source**	**Initial Nominal Resolution**	**Inclusion**	**Notes**
Topographic				
Elevation	GMTED2010^52^	250 m	+	
Slope	Derived from GMTED2010 above	250 m	+	
Northness	Derived from GMTED2010 above	250 m	+	Northness values inverted for southern hemisphere
Eastness	Derived from GMTED2010 above	250 m	+	
Terrain Ruggedness Index (TRI)	Derived from GMTED2010 above	250 m	+	Mean of absolute differences between a cell and its adjacent neighbors
Absolute Value of Latitude	Author generated using 500 m fishnet (ArcMap 10.1)	500 m	+	
Global Soils	Harmonized World Soils Database v. 1.2^53^	1 km	−	
Topsoil Clay Fraction (% wt.)	Harmonized World Soils Database v. 1.2^53^	1 km	−	
Topsoil Silt Fraction (% wt.)	Harmonized World Soils Database v. 1.2^53^	1 km	−	
Topsoil Sand Fraction (% wt.)	Harmonized World Soils Database v. 1.2^53^	1 km	−	
Topsoil Base Saturation (%)	Harmonized World Soils Database v. 1.2^53^	1 km	−	
Topsoil Bulk Density (kg/dm3)	Harmonized World Soils Database v. 1.2^53^	1 km	−	
Topsoil Calcium Carbonate (% wt.)	Harmonized World Soils Database v. 1.2^53^	1 km	−	
Topsoil Cation Exchange Capacity (cmol/kg)	Harmonized World Soils Database v. 1.2^53^	1 km	−	
Topsoil Organic Carbon (% wt.)	Harmonized World Soils Database v. 1.2^53^	1 km	−	
Topsoil pH (H2O) (−log(H+)	Harmonized World Soils Database v. 1.2^53^	1 km	−	
Reference Soil Depth	Harmonized World Soils Database v. 1.2^53^	1 km	−	
				
Climatic				
Annual Mean Temperature (Bio1)	WorldClim v. 1^54^	1 km	+	
Mean Diurnal Range (Mean of monthly (max temp - min temp)) (Bio2)	WorldClim v. 1^54^	1 km	−	
Isothermality (Bio2/Bio7) (* 100) (Bio3)	WorldClim v. 1^54^	1 km	−	
Temperature Seasonality (standard deviation *100) (Bio4)	WorldClim v. 1^54^	1 km	−	
Max Temperature of Warmest Month (Bio5)	WorldClim v. 1^54^	1 km	−	
Min Temperature of Coldest Month (Bio6)	WorldClim v. 1^54^	1 km	−	
Temperature Annual Range (Bio1-Bio2) (Bio7)	WorldClim v. 1^54^	1 km	+	
Mean Temperature of Wettest Quarter (Bio8)	WorldClim v. 1^54^	1 km	−	
Mean Temperature of Driest Quarter (Bio9)	WorldClim v. 1^54^	1 km	−	
Mean Temperature of Warmest Quarter (Bio10)	WorldClim v. 1^54^	1 km	−	
Mean Temperature of Coldest Quarter (Bio11)	WorldClim v. 1^54^	1 km	−	
Annual Precipitation (Bio12)	WorldClim v. 1^54^	1 km	+	
Precipitation of Wettest Month (Bio13)	WorldClim v. 1^54^	1 km	−	
Precipitation of Driest Month (Bio14)	WorldClim v. 1^54^	1 km	+	
Precipitation Seasonality (Coefficient of Variation) (Bio15)	WorldClim v. 1^54^	1 km	+	
Precipitation of Wettest Quarter (Bio16)	WorldClim v. 1^54^	1 km	−	
Precipitation of Driest Quarter (Bio17)	WorldClim v. 1^54^	1 km	+	
Precipitation of Warmest Quarter (Bio18)	WorldClim v. 1^54^	1 km	−	
Precipitation of Coldest Quarter (Bio19)	WorldClim v. 1^54^	1 km	−	
Potential Evapotranspiration per Hectare per Year (1950–2000)	CGIARCSI Global Aridity and PET Database^55,56^	1 km	+	
Indexed Annual Aridity (1950−2000)	CGIARCSI Global Aridity and PET Database^55,56^	1 km	+	
				
Vegetative				
Enhanced Vegetation Index (EVI)	Crowther *et al*^1^; Global Habitat Heterogeneity^57^	1 km	+	90th percentile of composited MOD13Q1 v. 5 (250 m) 16-day composites from 2001–2005, inclusive
Dissimilarity of EVI	Global Habitat Heterogeneity^57^	1 km	+	Difference in EVI between adjacent pixels
Contrast of EVI	Global Habitat Heterogeneity^57^	1 km	+	Exponentialy weighted difference in EVI between adjacent pixels
Uniformity (Angular Second Moment) of EVI	Global Habitat Heterogeneity^57^	1 km	+	Orderliness of EVI among adjacent pixels
Leaf Area Index	Global Land Cover Facility^58,59^	1 km	+	MOD09A1 derivation; 8-day composites from 2005, days 017 (s. hemisphere) and 193 (n. hemisphere)
Surface Reflectance Bands 1–7	Global Land Cover Facility^58,59^	500 m	−	MOD44C derivation; 32-day composites of 16-day MOD44C composites (Collection 3), beginning days 193 (2005 n. hemisphere) and 361 (2004 s. hemisphere)
Normalized Difference Vegetation Index (NDVI)	Derived from Surface Reflectance above	500 m	−	
				
Anthro.				
Urban/Built-up, and Cultivated and Managed Vegetation	Consensus Land Cover v. 1^57^	1 km	+	Including GlobCover; Classes 7 and 9
Human Appropriation of Net Primary Productivity	CIESIN: SEDAC^60^		−	
Global Human Footprint (1995–2004)	CIESIN: SEDAC^60^	1 km	−	v. 2
^1^Crowther, T. W. *et al.* Mapping tree density at a global scale. Nature. 525, 201–2015 (2015).				
^52^Danielson, J. J., and Gesch, D. B. 2011. Global Multi-resolution Terrain Dlevation Data 2010 (GMTED2010). U.S. Geological Survey, Reston, VA. (https://lta.cr.usgs.gov/GMTED2010).				
^53^FAO/IIASA/ISRIC/ISS-CAS/JRC, 2012. Harmonized World Soil Database (version 1.2). FAO, Rome, Italy and IIASA, Laxenburg, Austria. (http://webarchive.iiasa.ac.at/Research/LUC/External-World-soil-database/HTML/).				
^54^Hijmans, R.J., S.E. Cameron, J.L. Parra, P.G. Jones, and A. Jarvis. 2005. Very high resolution interpolated climate surfaces for global land areas. International Journal of Climatology, 25: 1965–1978. (http://www.worldclim.org/current).				
^55^Zomer, R. J., Trabucco, A., and van Straaten, O. 2007. A Global Analysis on the Hydrologic Dimensions of Climate Change Mitigation through Afforestation/Reforestation. International Water Management Institute, Research Report 101. (http://www.cgiar-csi.org/data/global-aridity-and-pet-database).				
^56^Zomer, R. J., Trabucco, A., Bossio, D. A., and Verchot, L. V. 2008. Climate change mitigation: A spatial analysis of global land suitability for clean development mechanism afforestation and reforestation. Agriculture Ecosystems and Environment, 126: 67–80 (http://www.cgiar-csi.org/data/global-aridity-and-pet-database).				
^57^EarthEnv: Global environmental layers for climate, ecosystem, and biodiversity research. (http://www.earthenv.org/).				
^58^Shunlin, L., and Zhiqiang, X. 2012. Global Land Surface Products: Leaf Area Index Product Data Collection(1985-2010). Beijing Normal University. (http://glcf.umd.edu/data/)				
^59^Xiao, Z., Liang, S., Wang, J., *et al.*, 2013. Use of general regression neural networks for generating the GLASS Leaf Area Index product from time series MODIS surface reflectance. IEEE Transactions on Geoscience and Remote Sensing. (http://glcf.umd.edu/data/).				
^60^Center for International Earth Science Information Network: Socioeconomic Data and Applications Center. (http://sedac.ciesin.columbia.edu/data/sets/browse).				

**Table 2:** Basic methods used to manage and pre-process spatial datasets.

**Spatial data pre-processing method**	**Description**
*Environmental Controls*	Used in conjunction with other operations to control geospatial products.
Processing extent	Used to process all datasets at a common extent to eliminate unexpected data loss around land mass peripheries prior to controlled masking.
Snap raster	Used to ensure all datasets of a common resolution had precise pixel-level spatial coincidence.
Projection	Used to ensure all datasets held a common coordinate system for processing (WGS84) and area-dependent tabulation (Interrupted Goode Homolosine).
*Construction*	
Mosaicking	Used to spatially mosaic datasets delivered in tiled format.
Nearest neighbor resampling	Used to unify raster cell size across datasets without introducing new data values.
Map algebra and geoprocessing tools	Used to produce derivative covariates (e.g., slope, aspect, etc.).
Spatial extraction/masking	Used to reduce all datasets to the smallest common extent prior to model fitting.
Spatial joining	Used to bind covariate values to coincident plot locations.

**Table 3:** Summary table showing the results of model validation.

**Terrestrial Biome**	***n***	**Pred. Mean**	**Obs. Mean**	**SD**	**Pred. Sum**	**Obs. Sum**	**SD Sum**
Boreal forests	1,116	98,157	94,459	1,168	109,542,928	105,416,204	1,303,045
Deserts	2,921	28,115	24,337	235	82,122,745	71,089,686	685,260
Flooded grasslands	55	47,691	50,576	2,894	2,623,006	2,781,658	159,169
Mangroves	—	—	—	—	—	—	—
Mediterranean forests	3,333	99,681	87,080	902	332,235,677	290,238,564	3,006,751
Montane grasslands	28	88,356	83,125	6,583	2,473,968	2,327,500	184,337
Temperate broadleaf	54,681	49,524	48,548	108	2,708,012,198	2,654,674,881	5,892,683
Temperate conifer	16,808	43,864	42,661	132	737,265,239	717,049,203	2,224,412
Temperate grasslands	3,415	30,406	28,215	264	103,835,175	96,353,092	900,426
Tropical coniferous	—	—	—	—	—	—	—
Tropical dry	17	48,525	30,938	4,083	824,925	525,938	69,415
Tropical grasslands	148	32,038	26,504	1,130	4,741,584	3,922,520	167,309
Tropical moist	1,017	80,839	77,722	795	82,212,834	79,043,004	808,476
Tundra	430	105,216	105,973	1,815	45,242,812	45,568,300	780,448
Total	83,969	752,410	700,137		4,211,133,091	4,068,990,550	
% Difference						3.5%	
*n*= number of withheld plots, ~20% of total; Pred. Mean=Predicted mean number of trees per pixel, post scaling, at locations of withheld 20% of plots; Obs. Mean=Observed mean number of trees per plot x 100, for withheld 20% of plots; s.d.=Standard deviation of pixel-level predictions at withheld 20% of plot locations; Pred. Sum=Sum of predicted number of trees per pixel at all plot locations within each biome, post scaling; Obs. Sum=Sum of observed number of trees per plot×100, for all plots within each biome; SD Sum=Standard deviation of predicted number of trees per pixel for all plot locations within each biome; % Difference=Percent difference in the total number of trees predicted (by pixel) and observed (plot×100) at all plot locations within each biome.							

**Table 4:** Summary table showing the number of field plots, estimates for the total number of predicted trees at those plots, and 95% confidence intervals on the estimates at biome and global scales.

**Terrestrial Biome**	**Number of field plots**	**Predicted number of trees (billions)**	**±2 s.d. (billions)**
Boreal forests	8,688	749.3	50.1
Deserts	14,637	53.0	2.9
Flooded grasslands	271	64.6	14.2
Mangroves	21	8.2*	0.3*
Mediterranean forests	16,727	53.4	1.2
Montane grasslands	138	60.3	24.0
Temperate broadleaf	278,395	362.6	2.9
Temperate coniferous	85,144	150.6	1.3
Temperate grasslands	17,051	148.3	4.9
Tropical coniferous	—	22.2*	0.4*
Tropical dry	115	156.4	63.4
Tropical grasslands	999	318.0	35.5
Tropical moist	5,321	799.4	24.0
Tundra	2,268	94.9	6.3
Global	**429,775**	**3041.2**	**96.1**

*Mangroves and Tropical coniferous biome predictions rely on models derived from Tropical moist and Temperate coniferous biomes, respectively. Given data limitations, figures associated with these biomes should be considered less reliable than those for the other biomes.
